# *TMPRSS2-ERG* activates NO-cGMP signaling in prostate cancer cells

**DOI:** 10.1038/s41388-019-0730-9

**Published:** 2019-02-04

**Authors:** Feng Zhou, Shuai Gao, Dong Han, Wanting Han, Sujun Chen, Susan Patalano, Jill A. Macoska, Housheng Hansen He, Changmeng Cai

**Affiliations:** 1 Department of Urology, The First Affiliated Hospital, School of Medicine, Zhejiang University, Hangzhou, Zhejiang 310003 China; 2 Center for Personalized Cancer Therapy, University of Massachusetts Boston, Boston, MA 02125 USA; 3 Dana-Farber/Harvard Cancer Center, Boston, MA 02215 USA; 4 Princess Margaret Cancer Center/University Health Network, Toronto, ON M5G1L7 Canada

**Keywords:** Transcription, Prostate cancer

## Abstract

The aberrant activation of the ERG oncogenic pathway due to the *TMPRSS2-ERG* gene fusion is the major event that contributes to prostate cancer (PCa) development. However, the critical downstream effectors that can be therapeutically targeted remain to be identified. In this study, we have found that the expression of the α1 and β1 subunits of soluble guanylyl cyclase (sGC) was directly and specifically regulated by ERG in vitro and in vivo and was significantly associated with *TMPRSS2-ERG* fusion in clinical PCa cohorts. sGC is the major mediator of nitric oxide (NO)-cGMP signaling in cells that, upon NO binding, catalyzes the synthesis of cGMP and subsequently activates protein kinase G (PKG). We showed that cGMP synthesis was significantly elevated by ERG in PCa cells, leading to increased PKG activity and cell proliferation. Importantly, we also demonstrated that sGC inhibitor treatment repressed tumor growth in *TMPRSS2-ERG*-positive PCa xenograft models and can act in synergy with a potent AR antagonist, enzalutamide. This study strongly suggests that targeting NO-cGMP signaling pathways may be a novel therapeutic strategy to treat PCa with *TMPRSS2-ERG* gene fusion.

## Introduction

Androgen receptor (AR), a steroid receptor transcription factor, plays a pivotal role in driving prostate cancer (PCa) initiation and progression [1]. The role of AR in PCa was further reinforced by the discovery of *ETS* gene rearrangements that lead to androgen-regulated expression of *ETS* family transcription factor proto-oncogenes [2–4]. The major form of the *ETS* rearrangements is the 5′ untranslated region of the androgen-regulated *TMPRSS2* (Transmembrane Protease Serine 2) gene fused to the exon 4 of *ERG* (V-Ets Erythroblastosis Virus E26 Oncogene Like) gene, resulting in the overexpression of transcriptionally active and N-terminal truncated ERG protein [2, 5]. This fusion is an early event in PCa initiation, as it can be detected in precursor prostatic intraepithelial neoplasia lesions (PIN) [6], and the fusion gene is also highly expressed in PCa tumors that have relapsed after androgen deprivation therapy (CRPC) [7]. The functions and activities of ERG have been previously studied and linked to cell mobility, invasion, EMT, and metastasis, and several downstream targets, including Myc, EZH2, Wnt, and Notch signaling pathways, have been reported [8–11]. ERG also cooperates with PI3K-AKT signaling to mediate PCa progression [12, 13]. In addition to its role as a direct transcription activator, ERG can function as a pioneer factor to regulate enhancer accessibility and reprogram the AR cistrome in PCa, leading to the expression of new AR-regulated genes such as *SOX9* [14, 15]. Although ERG plays a key role in PCa development, therapeutically targeting its expression or activity remains challenging. A recent study using peptidomimetic approaches to inhibit ERG signaling have shown promising results in pre-clinical models of PCa [16]. In this study, we took another approach and aimed to identify actionable downstream effector(s) of ERG that could provide novel therapeutic insights for patients harboring ERG alterations.

In addition to its role as an oncogenic factor in PCa and other cancers, ERG is a key transcription factor in endothelial cells and regulates functions such as angiogenesis and cell survival, thus driving endothelial cell lineage [17]. Therefore, the aberrant expression of ERG in PCa cells may lead to activation of pathways specifically related to these endothelial cell functions which may impact the initiation and progression of PCa. Through a comprehensive bioinformatic study to examine ERG-regulated genes, we have identified the α1 and β1 subunits (*GUCY1A1*, *GUCY1B1*) of soluble guanylyl cyclase (sGC) as major ERG-regulated endothelial genes that are also tightly associated with *ERG* expression in PCa patient samples. The α1 and β1 subunits heterodimerize to form the sGC protein, which is activated by nitric oxide (NO) and subsequently catalyzes the synthesis of cyclic guanosine monophosphate (cGMP), a critical second messenger that mediates many cellular functions of endothelial and smooth muscle cells, including ion channels, cell proliferation, and angiogenesis, through activating protein kinase G (PKG) and cGMP-gated ion channels [18]. We further showed that ERG can directly bind to the promoters of *GUCY1A1* and *GUCY1B1* and activate their transcription. Importantly, we found that ERG overexpression induced cGMP synthesis in vitro and in vivo, and that activated cGMP signaling promoted PCa cell proliferation. We then tested an available pharmacological sGC inhibitor on treating *TMPRSS2-ERG*-positive xenograft tumors and showed that inhibitor treatment alone or in combination with enzalutamide (an AR antagonist) can significantly suppress PCa tumor growth. Overall, this study suggests a new paradigm for effective PCa therapy for treating *TMPRSS2-ERG*-positive tumors.

## Results

### Identification of ERG-regulated genes that are clinically associated with *TMPRSS2-ERG* fusion in PCa

**Fig. 1:**
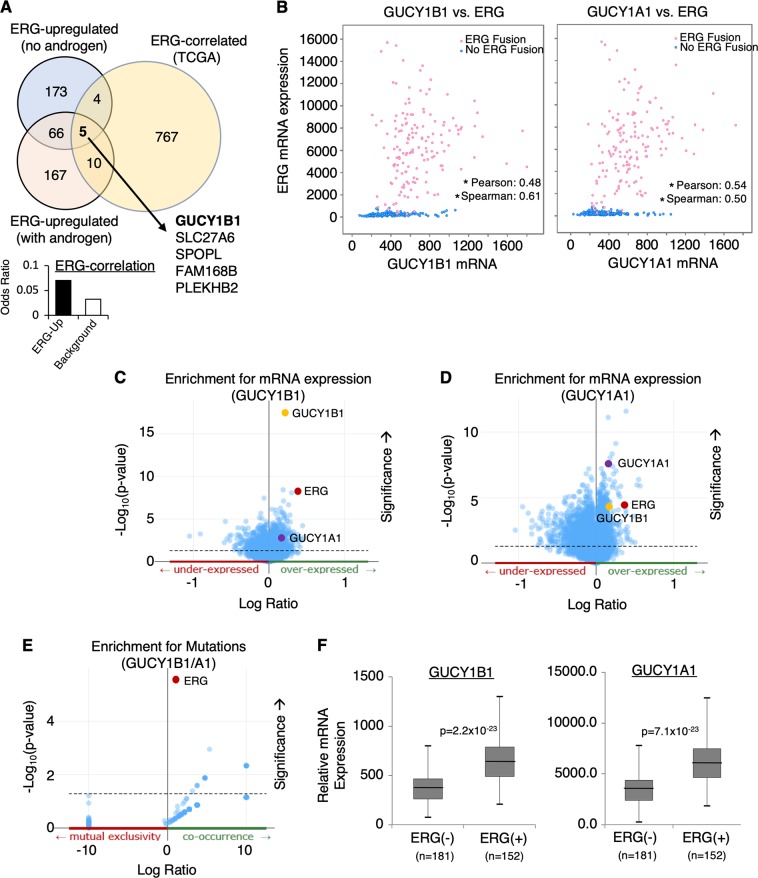
Identification of GUCY1A1 and GUCY1B1 as novel ERG-regulated genes that are clinically associated with TMPRSS2-ERG fusion in PCa. **a** ERG-upregulated genes were identified using Affymetrix microarray analyzes (Human Genome U133 Plus 2.0 Array) on VCaP cells stably infected with lentiviral shRNA against non-target-control or ERG and subsequently treated with ethanol or 10 nM DHT for 24 h. These genes were further examined for clinical correlation with ERG expression using TCGA PCa dataset. Note: The stable cells were hormone-starved for 3 days (d) before the treatment. **b** The mRNA expression correlation analyzes on GUCY1B1 vs. ERG or GUCY1A1 vs. ERG using TCGA PCa dataset (*N* = 333) from cBioPortal. The Pearson and Spearman rank correlation coefficient were shown and (*) indicates the *p*-value < 0.05. **c**, **d** Co-expressed genes associated with (**c**) GUCY1B1 or **d** GUCY1A1. **e** Co-exist mutations associated with the expressions of both GUCY1B1/A1. **f** Box plots for GUCY1B1 or A1 expression in ERG fusion-positive PCa vs. ERG fusion-negative PCa samples

To identify novel *TMPRSS2-ERG* regulated genes in PCa, we performed gene profiling analyzes on RNA extracted from VCaP cells (a *TMPRSS2-ERG*-positive, androgen-responsive PCa cell line) stably expressing shRNAs against ERG vs. non-target control (NTC) in the presence of androgens (to mimic the high androgen condition of primary PCa) or absence of androgens (to mimic the castrate condition during CRPC progression). These studies identified a subset of 248 genes upregulated by ERG in cells treated with DHT (dihydrotestosterone) and 248 genes in cells grown in hormone-depleted condition, with only 71 overlapping genes (Fig. 1a and Supplementary Figure 1). This significant change of ERG-regulated gene profile may be due to androgen stimulation of ERG expression or may indicate alterations of the ERG transcriptome by AR signaling.

To further identify ERG-regulated genes whose expression levels are clinically correlated with *TMPRSS2-ERG* expression in PCa patients, we carried out bioinformatic analyzes using TCGA primary PCa datasets (provided by cBioPortal) [19, 20]. Significantly, from this 71-gene subset we have then identified a group of five ERG-regulated genes whose expression levels are clinically correlated with *ERG* expression (~2-fold enrichment over background). The top ranked gene, *GUCY1B1*, encodes the β1 subunit of sGC, a critical enzyme that binds NO to catalyze the formation of cGMP [18]. The β1 subunit primarily heterodimerizes with the α1 subunit to form the sGC enzyme. Interestingly, the expression of the α1 subunit gene, *GUCY1A1*, also appeared to be ERG-regulated but slightly below the threshold of the analysis.

We found that the expression levels of *GUCY1B1* and *A1* were both positively correlated with *ERG* expression in the total PCa cohort (Fig. 1b) and *ERG* was among the top ranked genes whose expression was associated with increased expression of *GUCY1B1* and *A1* (Fig. 1c, d). We then examined the co-occurrence of *ERG* fusion gene with overexpression of *GUCY1B1/A1*. As seen in Fig. 1e, *TMPRSS2-ERG* fusion was the top ranked mutation that was significantly co-occurring with overexpression of *GUCY1B1*/*A1*. Finally, we determined whether *GUCY1B1/A1* were overexpressed in *ERG* fusion-positive PCa vs. negative PCa. As shown in Fig. 1f, the expression of both subunits was significantly higher in fusion-positive than in fusion-negative subset of patients. Similar results were also obtained from analyzes of Taylor PCa cohort [21] and Fraser PCa cohort [22] (Supplementary Figure 2A-C). As α2 (*GUCY1A2*) and β2 (*GUCY1B2*) subunits can also form sGC, we next examined whether the expression levels of these two genes are associated with *ERG* in TCGA cohort. As seen in Supplementary Figure 3, there was only weak correlation between *ERG* expression and *GUCY1A2* or *B2* expression, which was generally ~50–100 fold lower than the expression of *A1/B1*, suggesting that α2 and β2 are not major sGC subunits expressed in PCa cells. Together, these bioinformatic analyzes on public PCa datasets strongly suggest that the expression of sGC was clinically associated with *TMPRSS2-ERG*.

### ERG directly regulates the expression of sGC in PCa cells

**Fig. 2:**
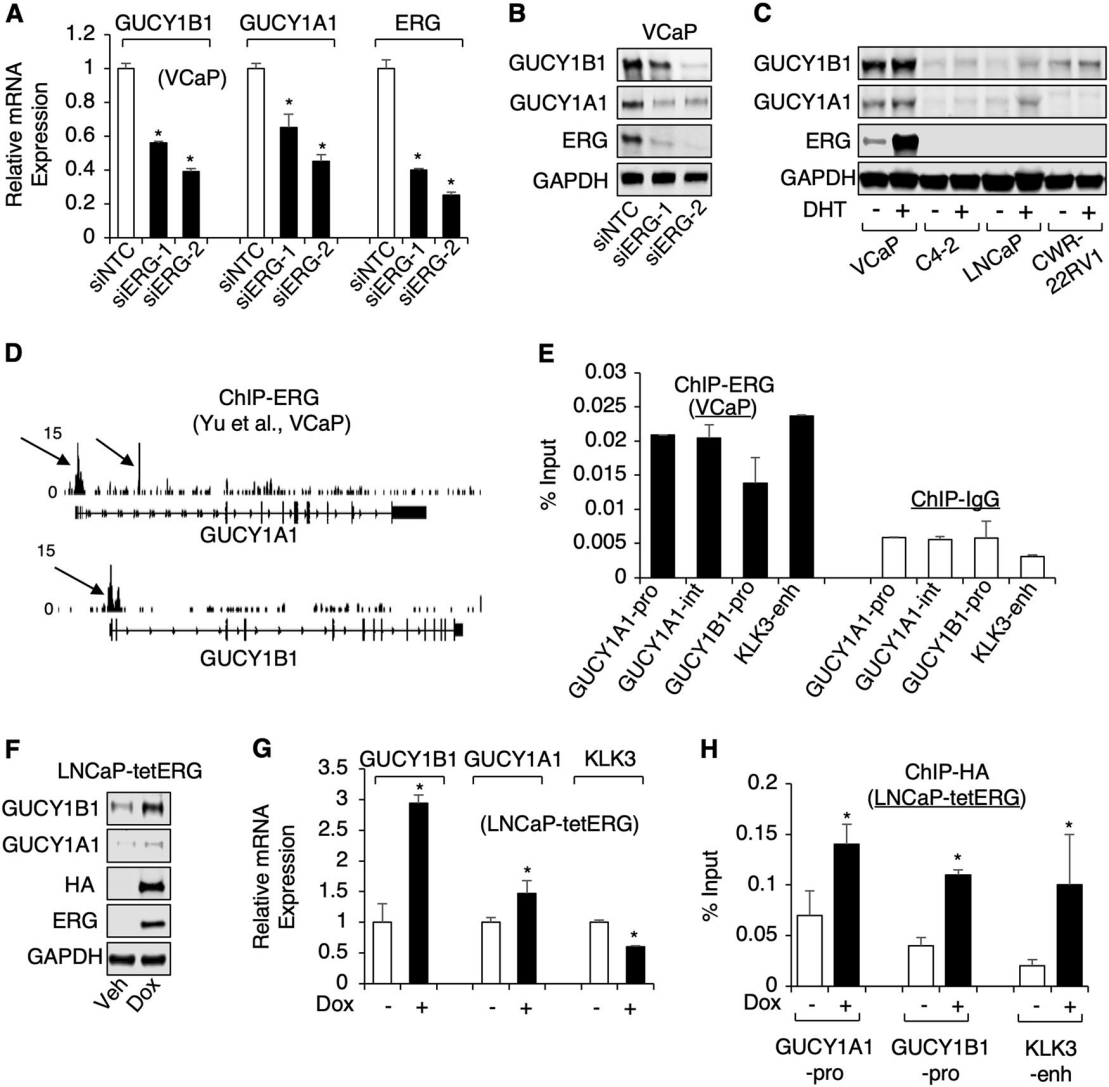
ERG directly regulates the expression of sGC in PCa cells. **a**, **b** VCaP cells transfected with siRNA against non-target-control (siNTC) or ERG (siERG1 or 2 targeting different regions of ERG) were subjected to **a** real-time RT-PCR or **b** immunoblotting. **c** Four PCa cell lines were hormone-starved for 3d and stimulated with DHT (10 nM) for 1d, and then subjected to immunoblotting. **d** ERG ChIP-seq binding peaks on gene loci of GUCY1B1/A1 in VCaP cells. **e** ChIP-qPCR for ERG binding at indicated gene loci. **f**, **g** LNCaP stable cells expressing doxycycline (dox)-inducible ERG (LNCaP-tetERG) were treated with dox for 2d, and then subjected to **f** immunoblotting or **g** real-time RT-PCR. **h** ChIP-qPCR for ERG binding (against HA tag) in LNCaP-tetERG cells after dox treatment for 2d

To validate the ERG regulation of sGC, we examined the expression of GUCY1A1 and B1 in VCaP cells transfected with ERG siRNA vs. NTC. As seen in Fig. 2a, b, both mRNA and protein expressions levels of the α1 and β1 subunits were significantly decreased by ERG silencing. We then compared the protein expression of sGC in fusion-positive VCaP cells vs. fusion-negative LNCaP, C4–2, and CWR22-RV1 PCa cell lines and found that the expression of both subunits was markedly higher in ERG-positive cells (Fig. 2c). The DHT treatment also increased the expression of α1 and β1 subunits in VCaP cells, presumably through stimulating *TMPRSS2-ERG* expression. Interestingly, the expression of sGC appeared to be androgen-induced even in ERG-negative LNCaP cells. It is now clear that LNCaP cells also harbor a chromosomal rearrangement of *ETV1* (ETS variant 1) locus that results in the androgen-regulation on ETV1 expression [4, 23]. Therefore, the increased expression of sGC by DHT treatment may be a result of androgen-induced expression of ETV1. Interestingly, the published ChIP-seq datasets on ETV1 and ETV4 chromatin binding in PCa cells [15, 24] indicate a possible ETV1 binding site in the 3′ untranslated region of *GUCY1A1* locus (no ETV1 binding was found on *GUCY1B1* gene locus) (Supplementary Figure S4A). Therefore, we next examined whether ETV1 can also regulate the expression of sGC. As seen in Supplementary Figure S4B, silencing *ETV1* did not decrease the expression of *GUCY1A1/B1*. Interestingly, the expression of *GUCY1A1* was modestly increased, suggesting that the ETV1 binding at *GUCY1A1* locus may function to repress its transcription. Nonetheless, this result indicated that the androgen regulation of sGC in LNCaP cells may occur through a distinct mechanism, by which AR can directly bind to the promoter of GUCY1A1 to activate its transcription as described previously [25].

We next determined whether ERG can directly bind to the *cis*-regulatory regions within the *GUCY1A1* and *B1* loci. From the analysis of a public ChIP-seq database [11], we have identified two ERG binding sites within the *GUCY1A1* gene and one site within the *GUCY1B1* gene (Fig. 2d) and all three sites contain multiple consensus ETS binding motives (not shown). Strong ERG binding was detected at all three sites using ChIP-qPCR of ERG in VCaP cells, and the binding levels were similar to the previously reported ERG binding at a *KLK3* enhancer [11] (Fig. 2e).

To further determine if ERG overexpression could increase the expression of sGC, we generated a LNCaP stable cell line with tetracycline-inducible ERG expression (HA tagged N-terminal truncated ERG) (LNCaP-tetERG). As seen in Fig. 2f, g, the protein and mRNA expression levels of the β1 subunit were significantly increased by doxycycline treatment, which only resulted in modest increased expression of the α1 subunit, suggesting β1 may be more strongly regulated by ERG. In contrast, the expression of a classic androgen-regulated gene, *KLK3*, was decreased by ERG, consistent with the previous report [11]. The induced ERG expression also increased ERG binding to the promoters of both sGC subunits (Fig. 2h). Overall, the above results highly suggest that the expression of sGC is specifically and directly regulated by *TMPRSS2-ERG* in PCa cells.

### ERG activates cGMP synthesis in PCa cells in vitro and in vivo

**Fig. 3:**
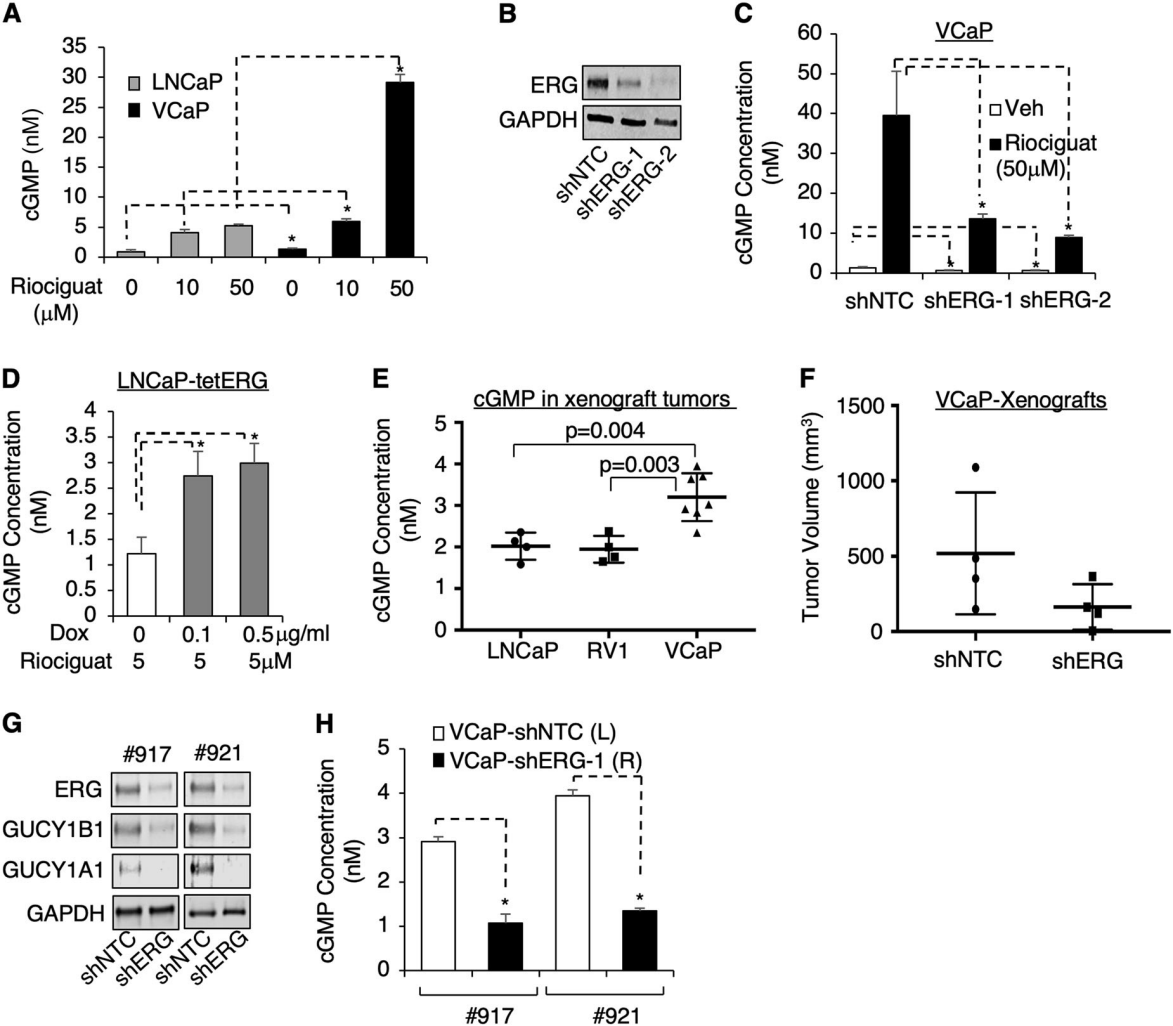
ERG activates cGMP synthesis in PCa cells in vitro and in vivo. **a** VCaP and LNCaP cells treated with 0, 10 or 50 μM riociguat for 24 h were subjected to the measurement of cellular cGMP level using ELISA kit. **b**, **c** VCaP cells stably infected with lentiviral shNTC or two independent shRNA against ERG (shERG-1,2) were **b** subjected to immunoblotting or **c** treated with or without riociguat (50 μM for 24 h), followed with cGMP ELISA assay. **d** LNCaP-tetERG cells pretreated with doxycycline for 3d and then treated with 50 μM riociguat for 24 h were subjected to cGMP ELISA assay. **e** Subcutaneous xenograft tumors derived from LNCaP (*N* = 4), CWR22-RV1 (RV1) (*N* = 4), or VCaP (*N* = 7) were established and then tumor biopsies were taken to examine the intratumoral cGMP level. **f**–**h** Equal amount of VCaP-shNTC or VCaP-shERG stable cells were subcutaneously injected into the left or right flank of the same mouse (*N* = 4) to allow xenograft tumor establishment. When the first xenograft reached ~1 cm in diameter, the group of mice were sacrificed and **f** tumor volumes were measured. Tumor biopsies from two mice were then taken for subsequent analyzes with **g** immunoblotting and **h** cGMP ELISA asay

The major activity of sGC in endothelial cells is to synthesize cGMP suggesting that ERG-regulated sGC expression may activate cGMP synthesis in PCa cells. To test this hypothesis, we first compared the level of cGMP in fusion-negative LNCaP cells vs. fusion-positive VCaP cells in response to an FDA-approved sGC activator, riociguat [26]. As seen in Fig. 3a, cGMP levels were modestly upregulated (~1.5-fold) in response to low-dose of riociguat and markedly elevated (~6-fold) in response to high-dose of riociguat in VCaP cells compared to LNCaP cells. We then sought to determine whether this increased response of cGMP synthesis in VCaP cells was dependent on ERG. As seen in Fig. 3b, c, stably silencing ERG expression resulted in ~50% decrease in basal cGMP levels and over 75% decrease in riociguat-induced cGMP levels (the fold induction of cGMP was also decreased from 32-fold to 21-fold and 13-fold), confirming the important role of ERG in activating sGC-mediated cGMP synthesis. A consistent result was also observed in LNCaP-tetERG cells showing increased cGMP synthesis by doxycycline-induced ERG overexpression (Fig. 3d).

Consistent with the expression of sGC and the level of cGMP synthesis in PCa cell lines, cGMP levels were significantly higher in VCaP-derived xenograft tumors than in xenograft tumors derived from fusion-negative LNCaP or CWR22-RV1 cells (Fig. 3e). To determine whether the high level of intratumoral cGMP in VCaP xenografts is ERG-dependent, we injected control and shERG VCaP stable cells into the same mouse at left and right flanks, respectively, and allowed the xenograft tumors to establish. As expected, the average tumor volume for the shERG group was smaller than the control group (Fig. 3f). We then selected two sets of matched tumor samples for further analyzes. Consistent with the in vitro studies, ERG silencing decreased the expression of both the α1 and β1 subunits (Fig. 3g), resulting in suppression of cGMP synthesis in vivo (Fig. 3h). Overall, the above in vitro and in vivo analyzes of cGMP synthesis strongly indicated that ERG-regulated transcriptional activation of sGC stimulates cGMP synthesis in PCa cells.

### Differential regulation of cGMP synthesis by sGC subunits in *TMPRSS2-ERG* positive PCa cells

**Fig. 4:**
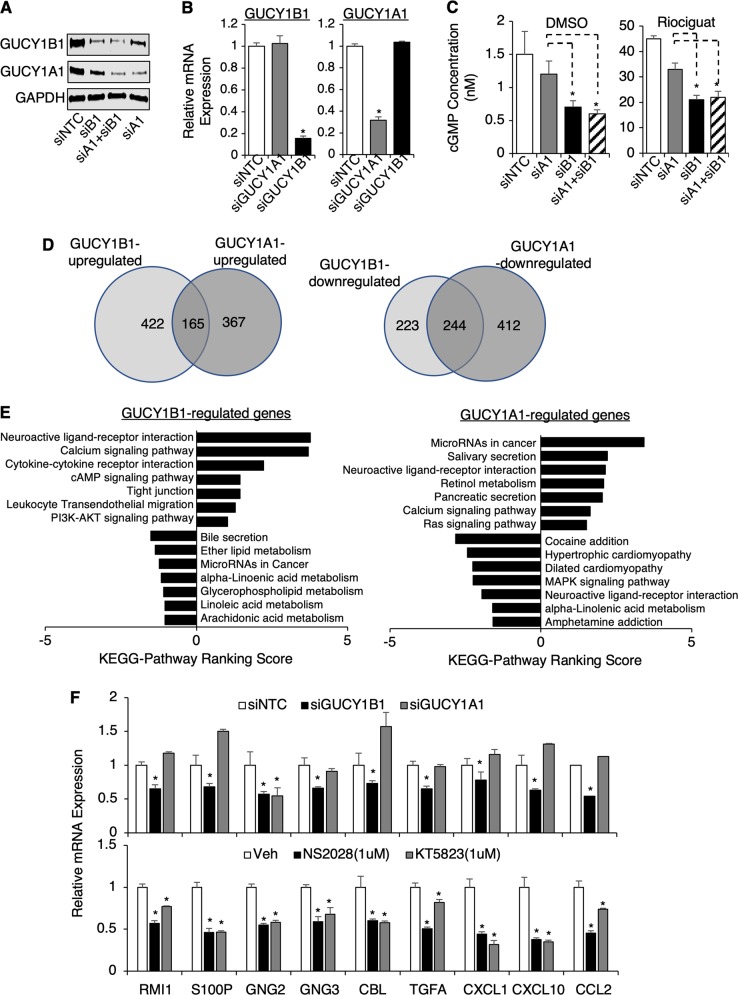
Global identification of sGC-regulated genes in TMPRSS2-ERG positive PCa cells. **a**–**c** VCaP cells transfected with siNTC or siRNA against GUCY1B1/A1 (siB1/siA1) individually or in combination were subjected to **a** immunoblotting, **b** real-time RT-PCR, or **c** cGMP ELISA assay with/out riociguat stimulation (12 h). **d**, **e** VCaP cells transfected with siNTC, siA1, or siB1 were subjected to RNA-seq analysis. **d** Venn diagram for overlapping or differentially regulated genes by GUCY1B1/A1 (cutoff 1.5-fold) and **e** KEGG pathway analyzes on GUCY1B1 or A1 regulated genes were presented. **f** A panel of GUCY1B1 upregulated genes were selected for subsequent validation. VCaP cells transected with siNTC, siA1 or siB1 (upper panel) or VCaP cells treated with NS2028 or KT5823 (1d) (lower panel) were subjected for real-time RT-PCR analyzes

We next sought to identify the downstream pathways and cellular functions of sGC-cGMP signaling in PCa cells. Although the activity of sGC requires heterodimerization of the α and β subunits, NO selectively binds to the HNOX (heme nitric oxide/oxygen binding) domain of β subunit. This led us to hypothesize that the level of the β1 subunit may be more critical to determine the rate of cGMP synthesis in PCa cells. To assess the differential contribution of sGC subunits to cGMP synthesis, an RNAi approach was used to specifically silence the expression of the α1 or β1 subunit in VCaP cells. Interestingly, silencing α1 gene expression decreased β1 protein but not its mRNA expression, and vice versa, suggesting that αβ dimerization may stabilize the protein expression of both subunits (Fig. 4a, b). More importantly, while silencing α1 only modestly decreased cGMP production, silencing β1 significantly suppressed cGMP synthesis in VCaP cells, and silencing both subunits did not further enhance this suppressive effect (Fig. 4c). These results suggest that the expression of β1 subunit is the rate limiting factor of cGMP synthesis in PCa cells.

To more broadly understand the cellular functions of the sGC subunits in PCa cells, we performed RNA-seq analyzes in VCaP cells treated with siRNAs against α1 or β1 vs. NTC. Differential gene expression analysis identified 587 β1-upregulated and 467 β1-downregulated genes (Fig. 4d, left panel), as well as 532 α1-upregulated and 656 α1-downregulated genes (Fig. 4d, right panel). Only 28% (165/587) of β1-upregulated genes were also upregulated by α1, but 52% (244/467) of β1-downregulated genes were similarly downregulated by α1. Consistent with the effect on cGMP synthesis, β1-upregulated genes enriched for many known functions of NO-cGMP signaling, including neuroactive ligand-receptor interaction, calcium signaling, cAMP signaling, tight junction, and leukocyte transendothelial migration (Fig. 4e, left panel). Importantly, β1-upregulated genes were also enriched for cancer-promoting pathways, such as cytokine receptor and PI3K-AKT pathways, the latter of which has been reported as the downstream signaling of sGC-cGMP [27–30]. Interestingly, the cytokine-cytokine receptor interaction was also one of the top enriched functions for ERG-upregulated genes (Supplementary Figure 5), suggesting that ERG may regulate specific cytokine signaling through sGC-cGMP pathway. Indeed, we have identified two cytokine genes, *CCL2* and *CXCL10*, whose expression levels were consistently decreased by either silencing of GUCY1B1 or ERG. Amongst them, CCL2 was previously shown to be a potent regulator of PCa cell migration and proliferation through activating PI3K/AKT pathway, suggesting that it may be a major downstream effector of sGC-cGMP in PCa cells [31]. Although α1-upregulated genes also enriched for neuroactive ligand-receptor interaction and calcium signaling, several distinctly enriched pathways were identified, including salivary/pancreatic secretion, retinol metabolism, cancer-related microRNAs, and Ras signaling (Fig. 4e, right panel). This finding of distinct α1 activities is consistent with our previous reports for possible cGMP-independent functions of sGCα1 in PCa cells [25, 32].

We next selected a panel of β1-upregulated genes representing enriched pathways for further validation. As shown in Fig. 4f, the expression levels of these genes were significantly suppressed by β1 silencing, but not by α1 silencing (see upper panel, except for *GNG2*), and were more significantly decreased by the treatment of sGC or PKG inhibitors (KT5823, which blocks both type I and II PKGs) (see lower panel), suggesting these tested genes are likely targets of the NO-cGMP pathway. Together, these results clearly demonstrated the predominant role of the β1 subunit in driving sGC-cGMP signaling in PCa cells.

Since the pathway analyzes indicated that the components of the PI3K-AKT pathway were regulated by β1, we then hypothesized that AKT may be activated by cGMP signaling in *TMPRSS2-ERG*-positive PCa cells. As seen in Supplementary Figure 6A, the sGC activator treatment increased AKT activity (based on Ser473 phosphorylation) while the sGC or PKG inhibitor treatment decreased AKT activity in VCaP cells. Moreover, the sGC or PKG inhibitor treatment also repressed AKT activity in ERG overexpressing LNCaP-tetERG cells (Supplementary Figure 6B). Overall, these results suggested that the PI3K-AKT pathway may be regulated by cGMP-mediated PKG activation in fusion-positive PCa cells.

### sGC-mediated cGMP signaling promotes cell proliferation in *TMPRSS2-ERG* positive PCa cells

**Fig. 5:**
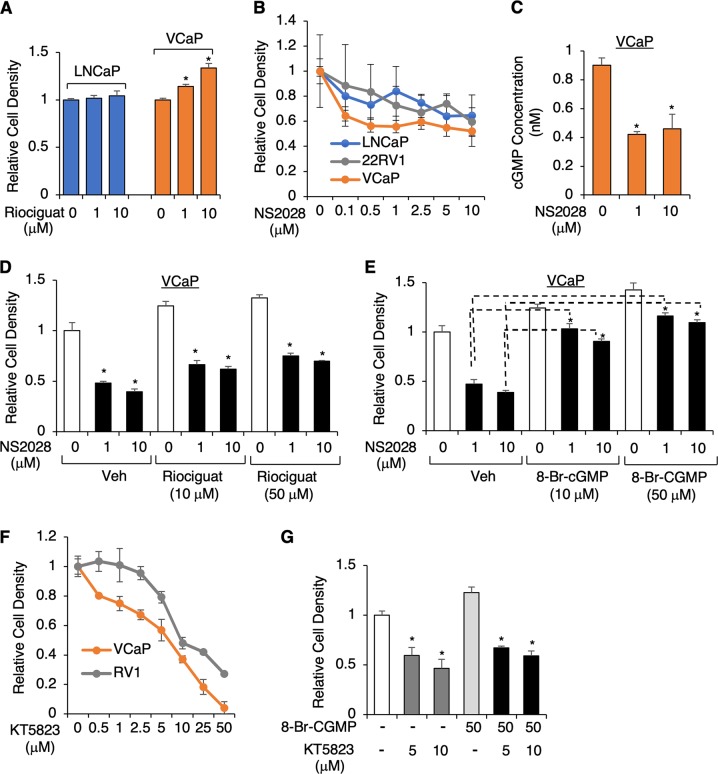
sGC-mediated cGMP signaling promotes cell proliferation in TMPRSS2-ERG-positive PCa cells. **a** VCaP and LNCaP cells treated with riociguat (0–10 μM) for 5d, and cell growth was measured by flow cytometry-based MUSE proliferation assay. **b** VCaP, CWR22-RV1, or LNCaP cells treated with NS2028 (0–10 μM) for 5d were examined for cell proliferation. **c** VCaP cells treated with NS2028 (0–10 μM) for 2d were subjected to cGMP ELISA assay. **d**, **e** VCaP cells pretreated with **d** riociguat (0–50 μM) or **e** 8-Br-cGMP (0~ 50 μM) were treated with NS2028 (0~ 10 μM) for 5d, followed by cell proliferation assay. **f** VCaP or CWR22-RV1 cells treated with KT5823 (0~ 50 μM) for 5d were examined for cell proliferation. **g** VCaP cells treated with KT5823 (0~ 50 μM) with/out 8-Br-cGMP (50 μM) for 5d were examined for cell proliferation

cGMP-stimulated proliferation of endothelial cells is an important driver of angiogenesis and this function has also been reported in other cell types and diseases [27, 28, 30, 33–35]. Therefore, we hypothesized that increased cGMP in *TMPRSS2-ERG*-positive PCa cells can promote tumor cell proliferation. We first examined whether the treatment of sGC activator promotes cell proliferation. As seen in Fig. 5a, riociguat treatment increased growth of fusion-positive VCaP cells but not fusion-negative LNCaP cells. We then assessed whether the treatment of an sGC inhibitor, NS2028, could decrease proliferation of fusion-positive cells. While two fusion-negative cell lines displayed modest responses to NS2028 treatment, with ~30% growth reduction at ~5–10 μM concentration, VCaP cells exhibited a stronger response, with ~45% growth reduction at only ~0.5–1 μM concentration (Fig. 5b), suggesting that the proliferation of ERG-positive PCa cells is more dependent on cGMP signaling. Consistent with the effect on proliferation, NS2028 treatment significantly blocked basal cGMP synthesis in VCaP cells (Fig. 5c). Furthermore, treating cells with 8-Br-cGMP (non-hydrolysable form of cGMP), but not sGC activator, rescued the cell proliferation from the sGC inhibitor-mediated growth inhibitory effect (Fig. 5d, e), indicating that the basal cGMP synthesis contributes to cell proliferation. A similar but more modest effect was also observed in experiments using another sGC inhibitor, ODQ (Supplementary Figure 7).

Since cGMP activates PKGs, we next assessed the effect of a PKG inhibitor (KT5823) on VCaP cell proliferation. As seen in Fig. 5f, g, KT5823 treatment more strongly inhibited the proliferation of VCaP cells compared to CWR22-RV1 cells, and adding additional 8-Br-cGMP did not rescue this growth inhibitory effect, suggesting that cGMP-mediated PKG activation contributes to VCaP cell proliferation.

cGMP has also been reported to regulate cell mobility and invasion [36]. Therefore, we next assessed whether sGC-CGMP signaling can affect PCa cell invasion. As seen in Supplementary Figure 8A, silencing sGC in VCaP cells did not alter their invasion capacity. Consistently, VCaP cell invasive capability was not affected by 8-Br-cGMP or PKG inhibitor (Supplementary Figure 8B). Overall, these results strongly indicated that activated cGMP-signaling mediates cell proliferation but not invasion in *TMPRSS2-ERG*-positive PCa cells.

### sGC inhibitor treatment decreases fusion-positive PCa tumor growth in vivo

**Fig. 6:**
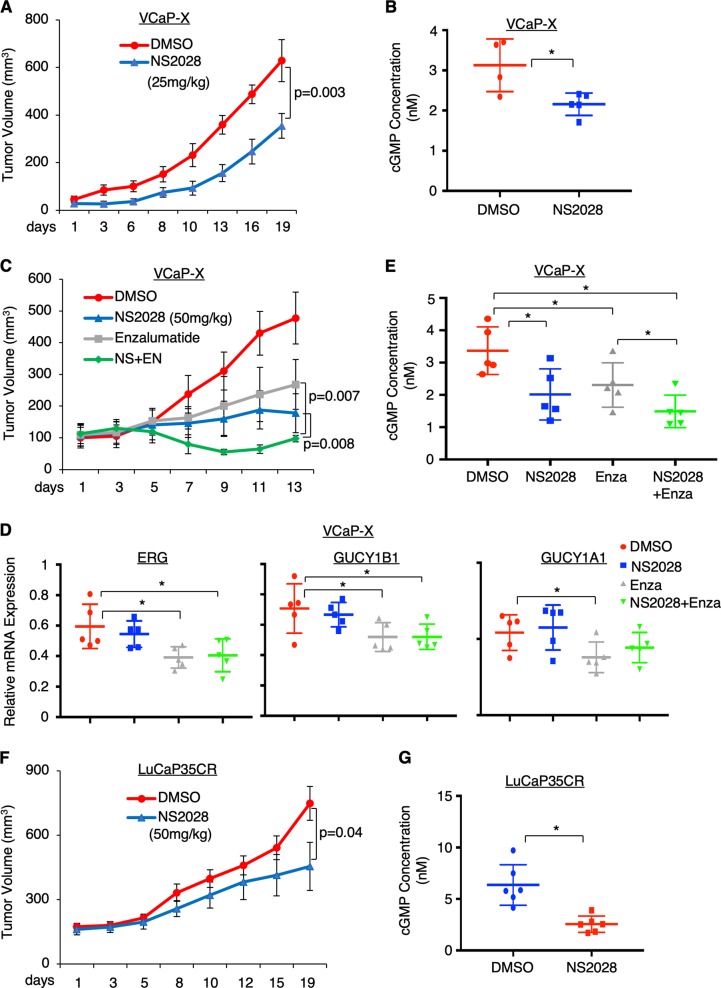
sGC inhibitor treatment decreases PCa tumor growth in vivo. **a**, **b** Male mice bearing VCaP xenograft tumors (*N* = 8) were treated with vehicle or NS2028 (25 mg/kg) through daily intraperitoneal injection. **a** Tumor growth was measured at indicated time points and **b** Intratumoral cGMP levels were measured in tumor biopsies at 19d. **c**–**e** Male mice bearing VCaP xenograft tumors (*N* = 6) were treated daily with vehicle, NS2028 alone (50 mg/kg), enzalutamide alone (10 mg/kg), or in combination. **c** Tumor growth was measured at indicated time points, **d** the expression of ERG, GUCY1B1, and GUCY1A1 was measured in tumor biopsies at 13d using qRT-PCR, and **e** Intratumoral cGMP levels were measured using the same group of biopsies. **f**, **g** Castrated male mice bearing LuCaP35CR xenograft tumors (*N* = 6) were treated daily with vehicle or NS2028 (50 mg/kg). **f** Tumor growth was measured at indicated time points and **g** intratumoral cGMP levels were measured in tumor biopsies at 19d

The above results strongly suggested that the sGC-mediated NO-cGMP pathway was activated in *ERG* fusion- positive PCa cells due to the transcriptional activation of sGC subunits. Therefore, directly targeting sGC may provide a novel strategy to treat *TMPRSS2-ERG*-positive PCa. NS2028 is a highly specific and potent sGC inhibitor and has been tested in animal studies [37, 38]. Therefore, we first assessed the efficacy of this inhibitor in a VCaP-derived xenograft model. As shown in Fig. 6a, mice bearing VCaP xenograft tumors treated with NS2028 (through daily intraperitoneal injection for ~3 weeks) demonstrated significantly reduced tumor growth. Consistent with the tumor regression, the cGMP synthesis and the expression of the identified cGMP-regulated genes were also decreased in NS2028-treated tumors (Fig. 6b and Supplementary Figure 9A). Since blocking cGMP pathway may affect tumor angiogenesis, we next examined whether NS2028 impacted the level of VEGF, a critical mediator and marker for angiogenesis. As seen in Supplementary Figure 9B, NS2028 did not significantly alter the VEGF levels in xenograft tumor biopsies, indicating that the anti-tumor effect of this inhibitor is not mediated through blocking tumor angiogenesis.

Androgen deprivation therapy (ADT) is the standard treatment for PCa patients, including those with *TMPRSS2-ERG*-positive tumors, and a more potent AR antagonist, enzalutamide, has been recently approved to treat PCa patients. Therefore, we next assessed whether NS2028 can be used in combination with enzalutamide to enhance the efficacy of ADTs. As shown in Fig. 6c, enzalutamide or NS2028 treatment alone both decreased tumor growth of VCaP-derived xenografts. The incomplete response to enzalutamide may be due to AR gene amplification in VCaP cells. Importantly, the combination treatment significantly enhanced the efficacy of single agent treatments, demonstrating the clinical potential of sGC inhibitor as a novel strategy to improve patient response to enzalutamide. While the enzalutamide treatment decreased the expression of ERG, and subsequently downregulated the expression of sGC subunits (Fig. 6d) and repressed intratumoral cGMP synthesis (Fig. 6e), the combination treatment more significantly reduced cGMP synthesis through further blocking sGC activity (Fig. 6e). Consistent with the effects on cGMP synthesis, the expression of cGMP-regulated genes was also further reduced by the combined treatment of NS2028 with enzalutamide (Supplementary Figure 10), indicating that the combination treatment is more effective in blocking NO-cGMP signaling.

To further examine whether sGC inhibition could be effective in treating *TMPRSS2-ERG*-positive CRPC, we also assessed the efficacy of sGC inhibitor treatment in a patient-derived (PDX) CRPC xenograft model, LuCaP35CR, which expresses AR and ERG and is resistant to castration [39, 40]. As seen in Fig. 6f, g, NS2028 significantly decreased the CRPC tumor growth and inhibited cGMP synthesis. The expression of cGMP-regulated genes was also decreased by sGC inhibition (Supplementary Figure 11A). However, VEGF expression was not significantly affected, consistent with the result from the study of VCaP xenograft (Supplementary Figure 11B**)**. Overall, these in vivo studies strongly suggest that sGC inhibitors have a strong therapeutic potential for treating *TMPRSS2-ERG*-positive primary PCa and CRPC.

## Discussion

Despite its function as an oncogenic transcription factor that drives the development of multiple cancers, including *ERG*-rearranged Ewing sarcoma [41] and PCa [2], ERG is predominantly expressed in normal endothelial cells and mediates endothelial cell differentiation, migration, proliferation, and angiogenesis [17, 41, 42]. Therefore, we hypothesized that the ERG-dependent endothelial cell-specific pathways may be activated in PCa cells to mediate oncogenic activities of ERG. To further identify novel ERG regulated genes/pathways that may mediate these endothelial functions, we performed a combined analysis using gene profiling data in a fusion-positive PCa cell line and patient data from public PCa datasets. Amongst the identified small subset of the clinically important ERG-regulated genes, we have discovered the α1 and β1 subunits of sGC, a well-known NO receptor that subsequently mediates cGMP synthesis, as major *TMPRSS2-ERG* targets in PCa cells. In vascular endothelial cells, sGC-cGMP pathway functions to promote angiogenesis and endothelial permeability [35, 43, 44]. In vascular smooth muscle cells, the sGC-cGMP signaling is well studied for its function in activating PKGs to reduce intracellular Ca^2+^ concentrations through phosphorylating multiple targets, resulting in smooth muscle relaxation [45]. The aberrant activation of cGMP pathways is linked to tumorigenesis in multiple cancers [34], and a recent study showed that the cGMP-dependent pathway can promote melanoma growth [33]. Significantly, our study shows that cGMP synthesis in PCa cells was strongly upregulated by *TMPRSS2-ERG* through transcriptionally activating sGC, and the increased cGMP synthesis promoted PCa cell proliferation. While this pro-proliferative activity of sGC-cGMP pathway might be, in part, due to the activation of PI3K/AKT signaling (see Fig. 4), which is an important downstream target of NO-cGMP pathway in regulating cell survival, migration and angiogenesis [27–30], other cGMP-regulated pathways, including calcium signaling and cytokine receptor activation, may also contribute to the PCa development.

Despite the recent development of peptidomimetic inhibitors of the *ERG* gene fusion [16, 46], a lack of targeting strategy for *TMPRSS2-ERG* signaling remains a major challenge in PCa treatment. In this study, we have discovered an important pro-proliferative pathway that is tightly regulated by *TMPRSS2-ERG*, providing a novel strategy to target ERG activity through blocking the NO-cGMP pathway. These strategies included directly targeting sGC by sGC inhibitors and blocking downstream PKG activation by PKG inhibitors, and we have demonstrated the clinical potential of these inhibitor treatments in vitro and in vivo in *TMPRSS2-ERG*-positive PCa (see Figs. 5 and 6). ADTs are the standard of care in treating metastatic PCa but most cancer will progress to CRPC within a few years with restored AR activity and re-expression of AR-regulated genes, including ERG. Therefore, co-targeting AR activity and ERG-regulated NO-cGMP pathway may provide a therapeutic approach to prevent or delay the recurrence of CRPC. Using a VCaP-derived xenograft model, we have demonstrated that the sGC inhibitor can synergize with a more aggressive ADT agent, enzalutamide, to reduce PCa tumor proliferation. Furthermore, using an ERG-positive PDX model we demonstrated that sGC inhibitor treatment was also effective in reducing tumor growth of CRPC. One of the possible adverse effects of using sGC inhibitor in PCa patients is that the treatment may induce hypertension. However, this may be prevented by co-treating the anti-hypertensive drugs that are not acting through NO-cGMP pathways. Nonetheless, these results strongly suggest that treatments targeting NO-cGMP pathway could emerge as a novel therapeutic strategy to treat *TMPRSS2-ERG*-positive PCa.

Another important pathway that regulates the intracellular cGMP levels is mediated by phosphodiesterase enzymes (PDEs), which are directly activated by binding of cGMP and then catalyze the degradation of cGMP to provide a feedback regulation on turning off NO-cGMP signaling [45]. Inhibiting these PDEs resulted in the accumulation of intracellular cGMP that promotes smooth muscle relaxation and other endothelial functions. PDE inhibitors, particularly PDE5 inhibitors such as sildenafil, vardenafil, and tadalafil, have been widely used in treating men with erectile dysfunction and other vascular diseases [47]. However, clinical use of PDE5 inhibitors have been linked to increased cancer risk. For example, sildenafil use was reported to significantly associate with an increased risk of developing melanoma, possibly through cGMP-pathway activated MAPK signaling [33, 48]. In PCa, a recent clinical study reported that use of PDE5 inhibitors may be clinically associated with increased biochemical recurrence after radical prostatectomy [49] although contradictory results were reported in other studies [50]. Our study has provided a possible mechanistic basis for the observed increased risk of PCa with use of PDE5 inhibitors, as these inhibitors can activate cGMP pathways to promote PCa cell proliferation. Our study also suggests that this potential PCa risk may be further increased in the subset of patients harboring *TMPRSS2-ERG* fusion, as the cGMP synthesis rate was significantly higher due to the transcriptional activation of sGC by ERG overexpression (see Fig. 3). Similar to PDE inhibitors, sGC activators, which are also widely used in treating diseases including cardiovascular disease and urinary track disorders [51], may also potentially increase the PCa risk due to the activation of cGMP pathways. Future studies are clearly needed to assess whether or how the use of PDE5 inhibitors or sGC activators affect PCa risk in the *TMPRSS2-ERG*-positive subgroup of patients.

In addition to showing ERG regulation on sGC expression, we have also previously shown that the expression of the α1 subunit of sGC was specifically upregulated by androgen treatment in LNCaP cells [25]. A similar result was also seen in this study (see Fig. 2c). This regulation is possibly due to the direct binding of AR on *GUCY1A1* promoter [25] but not through androgen-induced expression of ETV1 in LNCaP cells (see Supplementary Figure 4). In contrast to the ERG regulation on the expression of both subunits, AR only activates the transcription of the α1 subunit [25], although the protein expression of β1 subunit may be indirectly increased through possible protein stabilization consequent αβ dimerization (see Fig. 4a). Interestingly, we previously reported that induced expression of the α1 subunit promoted a cGMP-independent pro-proliferative activity in LNCaP cells, possibly due to the downregulation of p53 activity [25, 32]. Our current study was consistent with this observation, showing that the expression of the β1 subunit played a predominant role in driving cGMP synthesis in fusion-positive PCa cells and the α1 subunit may have distinct cGMP-independent activities such as activating cancer-promoting miRNA and Ras signaling (see Fig. 4e). Importantly, we have developed a class of peptides that specifically blocked the cGMP-independent activity of the α1 subunit of sGC and show that the peptide treatments induced cell apoptosis in vitro and in vivo [52, 53]. Based on the current study, we anticipate that these peptide treatments may synergize with the sGC/PKG inhibitors in treating *TMPRSS2-ERG*-positive PCa. Future in vitro and in vivo studies are clearly required to determine the efficacy of these combination treatments.

In summary, we have discovered that sGC is a novel transcriptional target of *TMPRSS2-ERG* in PCa cells, and showed that the sGC-mediated NO-cGMP pathway was a critical downstream effector of ERG in promoting cancer cell proliferation and tumorigenesis. Therapeutic treatments that directly target sGC-cGMP pathway may become a novel strategy to specifically treat PCa patients with *TMPRSS2-ERG* fusion tumors. We strongly believe that with the future development of sGC/PKG inhibitors these promising preclinical findings can be rapidly translated into PCa therapies.

## Materials and methods

### Cell lines

The VCaP, LNCaP, and CWR22RV1 cell lines were authenticated every year based on short tandem repeat (STR) profiling. VCaP and LNCaP cells (including LNCaP-derived stable lines) were cultured in RPMI-1640 with 10% FBS. CWR22RV1 cells were cultured in RPMI-1640 with 10% CSS (charcoal-stripped FBS). The LNCaP-tetERG stable cell line was maintained in RPMI-1640 with 10% tetracycline-free FBS. For experiments requiring androgen treatment, cells were hormone-depleted in 5% CSS for 2–3 days prior to androgen-treatments.

### Chromatin immunoprecipitation (ChIP)

To prepare ChIP-seq, PCa cells were formalin fixed, lysed, and sonicated to allow chromatin to break into 500–800 bp fragments, followed by immunoprecipitation. The SYBR Green based quantitative real-time PCR (qPCR) was subsequently carried out using the QuantStudio 3 Real-time PCR system (Thermo Fisher Scientific). The primers are listed as following: *GUCY1A1*-pro: forward, 5′- CAAGGAGGACTGTCTGGGAG-3′, reverse, 5′- GGTCGCTCATGTCTACCTGT-3′; *GUCY1A1*-int: forward, 5′- CGAGGGAGAGAGAGAGGAGA-3′, reverse, 5′-AGTTCCTTCAAGAGCTGGCT-3′; *GUCY1B1*-pro: forward, 5′- CTCTGCCTGGGTCCCTTC-3′, reverse, 5′-CACTTACCATGGTGTCTGCA-3′; *KLK3*-enh: forward, 5′-GCCTGGATCTGAGAGAGATATCATC-3′, reverse, 5′-ACACCTTTTTTTTTCTGGATTGTTG-3′.

### RT-PCR and RNA-seq

RNA was extracted with TRIzol Reagent (Invitrogen) according to the manufacturer′s protocol. The gene expression was examined using qRT-PCR analyzes (Taqman based one-step RT-PCR reagents) and results were normalized to co-amplified GAPDH. The primers and probes are listed as following: *ETV1* (Hs00951951_m1), *GUCY1A1* (Hg05088657_m1), *GUCY1B1* (Hg05103896_m1), *RMI1* (Hs01857117_s1), *S100P* (Hs00195584_m1), *GNG2* (Hs00828232_m1), *GNG3* (Hs00360009_g1), *CXCL1* (Hs00236937_m1), *CXCL10* (Hs00171042_m1), *CCL2* (Hs00234140_m1), *CBL* (Hs01011446_m1), *TGFA* (Hs00608187_m1), *GAPDH* (Hs00171834_m1) (purchased from Applied Biosystems at Thermo Fisher); *KLK3*: forward, 5′-GATGAAACAGGCTGTGCCG-3′, reverse, 5′-CCTCACAGCTACCCACTGCA-3′, probe, 5′-FAM-CAGGAACAAAAGCGTGATCTTGCTGGG-3′; *ERG*: forward, 5′-CAAGTAGCCGCCTTGCAAA-3′; reverse, 5′-GCTCCAGGAGGAACTGCCA-3′; probe, 5′-FAM-CCAGGCAGTGGCCAGATCCAGC-3”. For RNA-seq analysis, RNA was purified using RNeasy Mini Kit (Qiagen). TruSeq® Strnd Total RNA LT (Illumina) was used for library construction, and sequencing was performed on HiSeq 2000 Illumina Genome Analyzer. The differential gene expression analysis was performed using TopHat pipeline on Galaxy.

### Immunoblotting

For immunoblotting, RIPA buffer with protease inhibitors was used to extract protein from cells and anti-GUCY1A1 (Cayman), anti-GUCY1B1, anti-ERG (Santa Cruz), anti-AKT, anti-pAKT(S473), anti-VEGF (Cell Signaling), anti-HA (Sigma), anti-β-actin (Abcam), or anti-β-tublin (Upstate) antibodies were used. Gels shown are representative of at least three independent experiments.

### RNAi and small molecule treatments

siRNA against ERG, GUCY1A1, GUCY1B1, NTC were purchased from Dharmacon and transfected into cells using lipofectatimine 2000 (Thermo Fisher). VCaP cells were infected with lenti-virus containing shRNAs against ERG or NTC (Dharmacon) and mixed with polybrene (Millipore) following manufacture’s protocol, and further selected in medium containing puromycin. The small molecule treatments were listed as following: ODQ (Sigma), riociguat (Selleck), NS2028 (Cayman), KT5823 (Cayman) and 8-Br-cGMP (Sigma).

### Cell proliferation assay

PCa cells were seeded with 10% FBS. After treatments, cells were collected and fixed with ice cold 70% ethanol for 3 h, followed by staining with Muse Count & Viability Assay kit for 30 min and cell counting by Muse® Cell Analyzer, a compact flow cytometry system (EMD Millipore).

### Xenografts

Approximately 2 million VCaP cells (mixed with 50% Matri-gel) were injected in the flanks of male SCID mice to develop xenograft tumors. LuCaP35CR xenograft was established in the flanks of castrated male SCID mice by transplantation. Tumor volume was measured by manual caliper. The necrotic tumor tissue was excluded from the experiments by examining the frozen samples.

### cGMP ELISA

Cells or tissue were treated with 0.1 M HCl (1 × 106 cells per mL of HCl or tissue in 10X volumes of HCl) for 10 min to ensure uniform lysis (incubating for additional 10 mins, if necessary) and then subjected to cGMP ELISA assay using a direct cGMP ELISA KIT (Enzo) following the manufacturer’s protocol.

### Statistical analysis

The results in bar graphs are presented with mean + SD (standard deviation), which are calculated based on at least 3 biological repeats. Student’s *t*-test was used to compare treatment vs. control or otherwise as indicated. Statistical significance of the difference was determined based on *p*-value < 0.05 (*). Immunoblotting pictures are representative of at least three experiments. For animal studies, the sample size of mouse xenograft studies was estimated based on power analysis and the animals were randomized into two (single agent treatment) or four (combination treatment) experimental groups. The blinding was required for tumor volume measurement and the student’s *t*-test was used to compare the mean tumor volume (presented by mean ± SD) at the indicated time points for animal studies.

### Accession numbers

The GEO accession for *GUCY1A1/B1* siRNA RNA-seq data is GSE114738.

## Supplementary information

Supplementary Figures and Legends
