# Supplementary material for: TMPRSS2-ERG activates NO-cGMP signaling in prostate cancer cells
Source: Oncogene. 2019 Feb 4;38(22):4397–411. doi: 10.1038/s41388-019-0730-9 (PMC6542710; doi:10.1038/s41388-019-0730-9)
Supplement: Supplementary file 1 — Supplementary Figures and Legends [file 41388_2019_730_MOESM1_ESM.pdf]

SUPPLEMENTARY FIGURES:

Gene	FC (Veh)	FC (DHT)	Gene	FC (Veh)	FC (DHT)
ID1	3.0	8.6	PCGF6	1.5	1.9
ID3	1.8	5.1	KDELR3	1.8	1.9
WDR33	3.5	3.4	SLC3A1	1.7	1.8
MYLK	2.7	3.4	SLPI	1.6	1.8
APOH	1.9	3.2	RAB31	1.9	1.8
SERPINA3	3.9	3.2	GNE	1.6	1.8
NPY	1.7	3.1	CAMK2D	1.5	1.8
SLC40A1	2.1	3.1	BIRC3	2.9	1.8
RARRES3	2.7	3.1	RBMS1	1.7	1.8
RGS2	2.4	2.8	NAT1	1.7	1.8
SLC27A6	1.6	2.5	RBP7	1.8	1.7
CRYZ	2.0	2.4	RIN2	1.6	1.7
NOSTRIN	1.8	2.4	HNMT	2.3	1.7
EMP1	2.5	2.3	ERRFI1	2.0	1.6
CCL2	4.7	2.3	OAS1	1.9	1.6
GPX8	2.2	2.3	CSTA	2.1	1.6
FAM114A1	1.8	2.2	HIST1H1C	1.6	1.6
SHISA2	2.1	2.1	ISG20	1.8	1.6
PSMB8	1.8	2.1	KCNMB3	1.6	1.6
VAMP8	1.8	2.0	MYO10	1.6	1.6
CRISPLD1	2.1	2.0	UBE2L6	1.6	1.6
TSPAN8	1.9	2.0	RNF182	1.7	1.6
HEPACAM2	2.2	2.0	GPX2	2.0	1.6
FAM129A	2.4	2.0	SNX7	1.6	1.6
RELN	2.2	2.0	BIN1	1.5	1.6
PGC	2.0	2.0	HGF	4.4	1.6
AKAP12	2.2	2.0	NUDT11	2.0	1.6
SPOPL	1.6	2.0	KLHL5	1.5	1.6
ARHGAP28	2.6	1.9	TMSB15A	2.1	1.5
HAO1	1.8	1.9	SELM	1.5	1.5
ALDH1A1	2.3	1.9	DSCR6	1.7	1.5
OPTN	1.8	1.9	GUCY1B1	1.5	1.5
GATA6	1.7	1.9	FAM168B	1.6	1.5
DNAJC22	1.7	1.9	AMACR	1.8	1.5
ACOX2	1.9	1.9	LITAF	1.8	1.5
PLEKHB2	1.8	1.9			

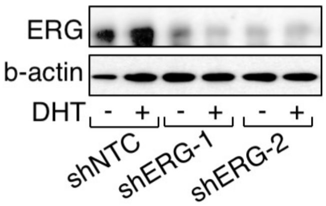

Supplementary Figure 1. Identification of genes that were ERG-regulated under both hormone-depleted and androgen-stimulated conditions. Affymetrix gene expression array analyses were performed in VCaP cells stably infected with lentiviral shRNA against non-target-control or ERG (shERG-2 line was used) and subsequently treated with ethanol or 10nM DHT for 24h. The overlapping ERG-regulated genes (71 genes) in both conditions were further identified and listed (cutoff 1.5-fold).

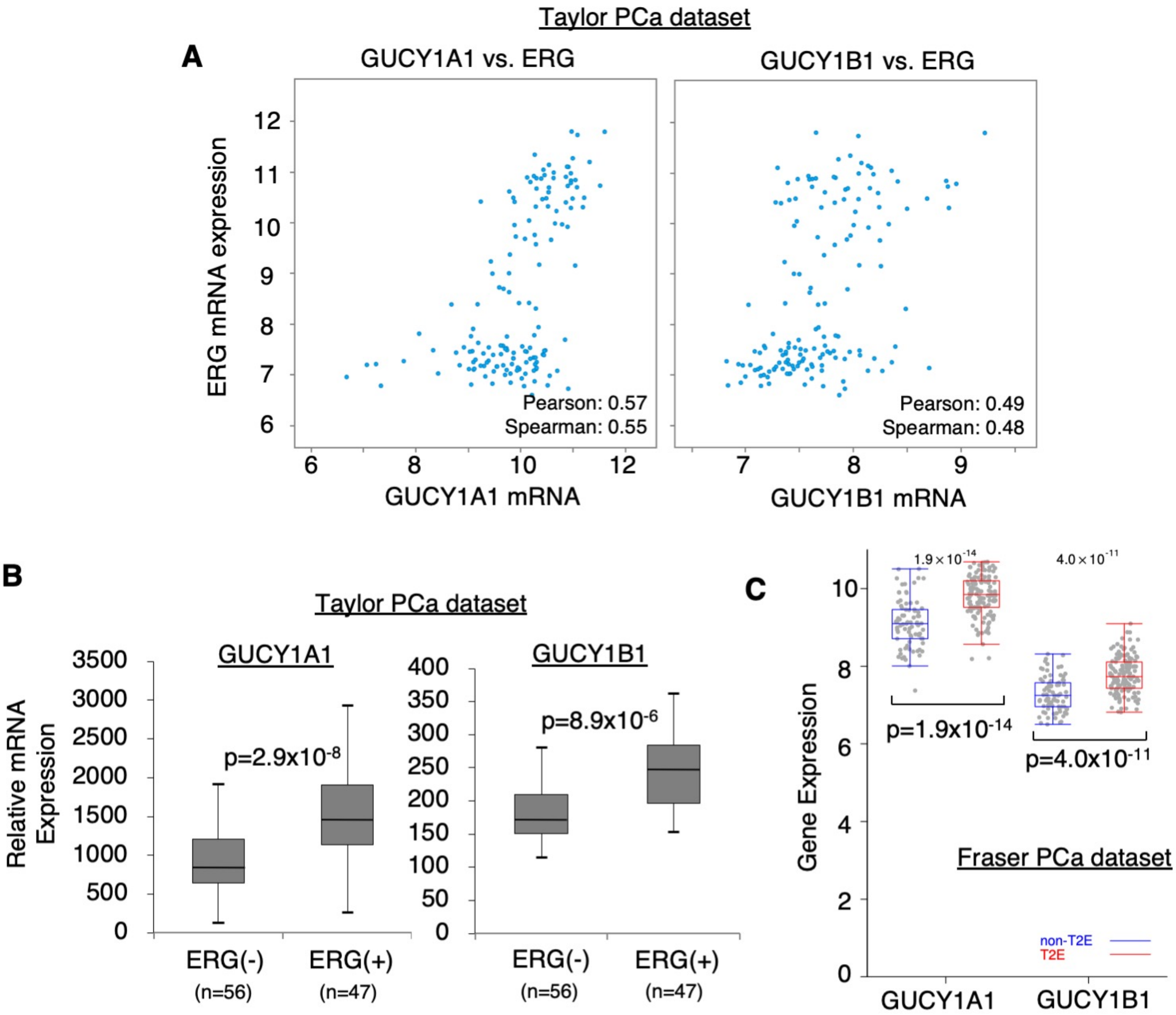

Supplementary Figure 2. The GUCY1A1 and B1 expressions were correlated with ERG expression in Taylor and Fraser PCa datasets. (**A**) The mRNA expression correlation analyses on GUCY1A1 versus ERG or GUCY1B1 versus ERG using Taylor PCa dataset (N=103). (**B**) Box plot for GUCY1A1 or B1 expression in ERG-negative versus ERG-positive PCa samples. (**C**) Box plot for GUCY1A1 or B1 expression in ERG-negative versus ERG-positive PCa samples using Fraser PCa dataset (N=200).

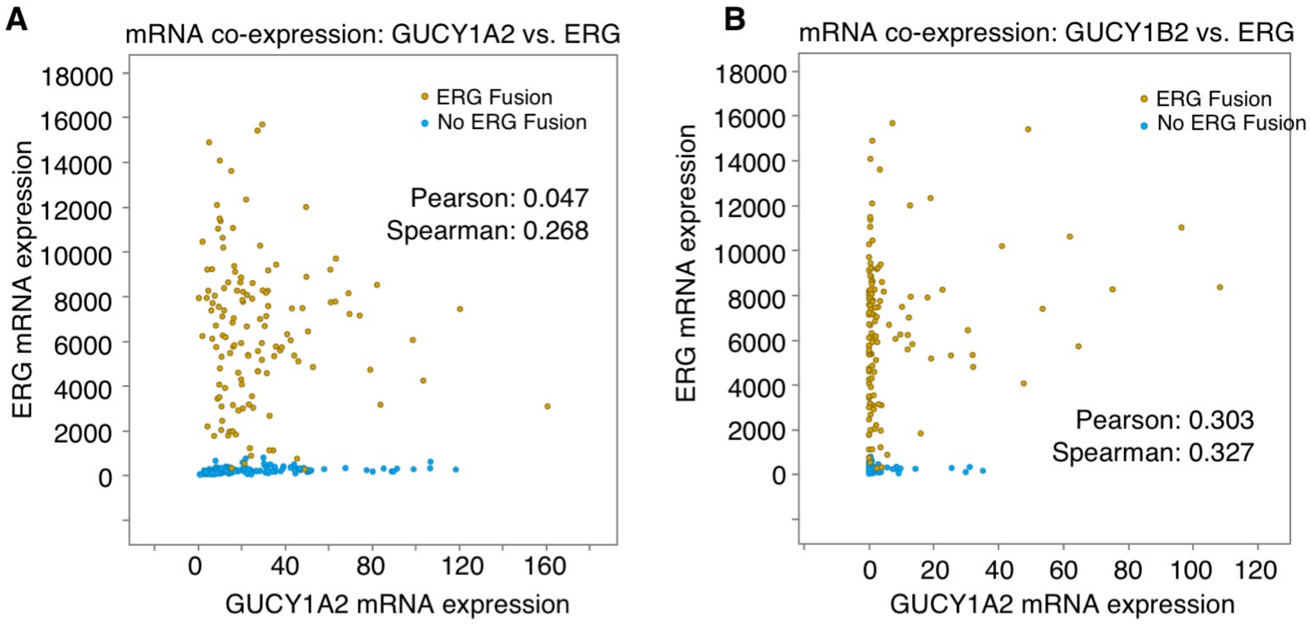

Supplementary Figure 3. The GUCY1A2 or B2 expression was very low and not significantly associated with ERG. (**A**, **B**) The mRNA expression correlation analyses on (A) GUCY1A2 versus ERG and (B) GUCY1B2 versus ERG using TCGA PCa dataset (N=333) from cBioPortal.

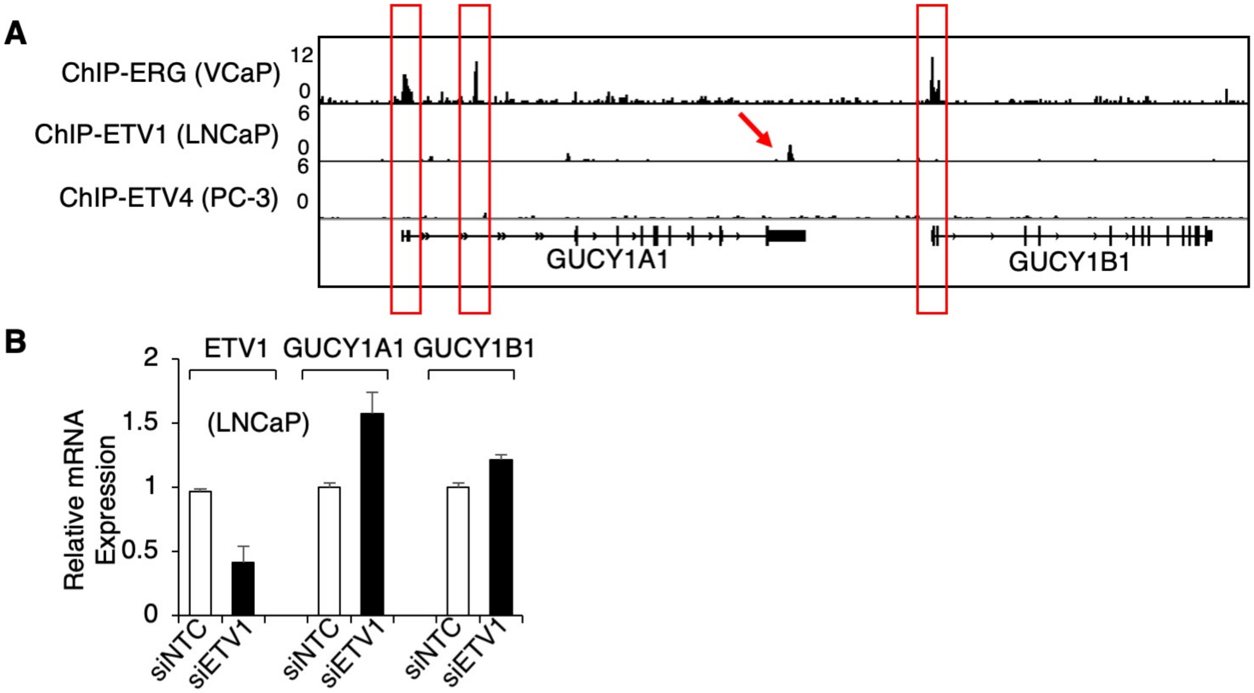

Supplementary Figure 4. The GUCY1A1 or B1 expression was not decreased by silencing ETV1. (**A**) ChIP-seq of ERG (in VCaP cells), ETV1 (in LNCaP cells), or ETV4 (in PC3 cells) at GUCY1A1/B1 gene locus. **(B)** LNCaP cells transfected with siNTC or siETV1 were subjected to real-time RT-PCR.

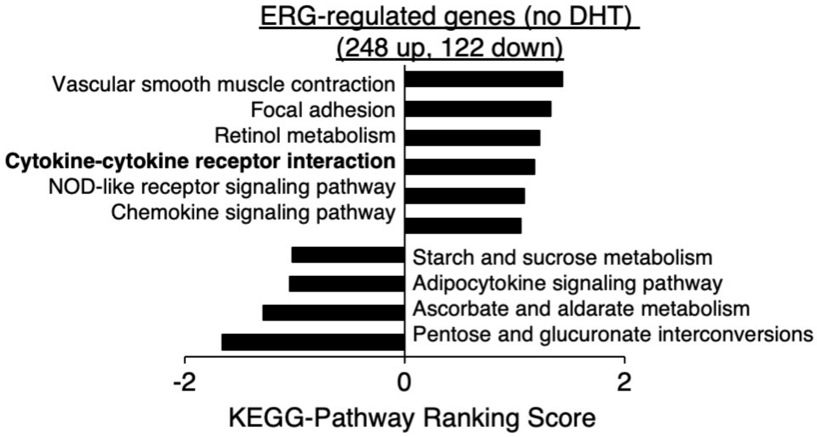

Supplementary Figure 5. ERG upregulated genes enriched for cytokine-cytokine receptor interaction. KEGG pathway analyses on ERG up- or down-regulated genes (results from Affymetrix microarray analysis on VCaP cells stably infected with lentiviral shRNA against non-target-control or ERG).

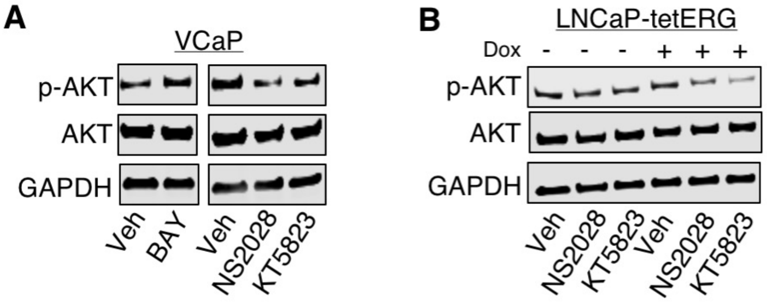

Supplementary Figure 6. sGC-cGMP pathway activated AKT in ERG-positive PCa cells. (**A**) VCaP cells treated with BAY, NS2028, or KT2853 (30min) were subjected for immunoblotting for Ser473-phosphorylated ATK (p-AKT) and total AKT. (**B**) LNCaP-tetERG cells (pretreated doxycycline for 2d) were then treated with BAY, NS2028, or KT2853 for 1d, and followed by immunoblotting.

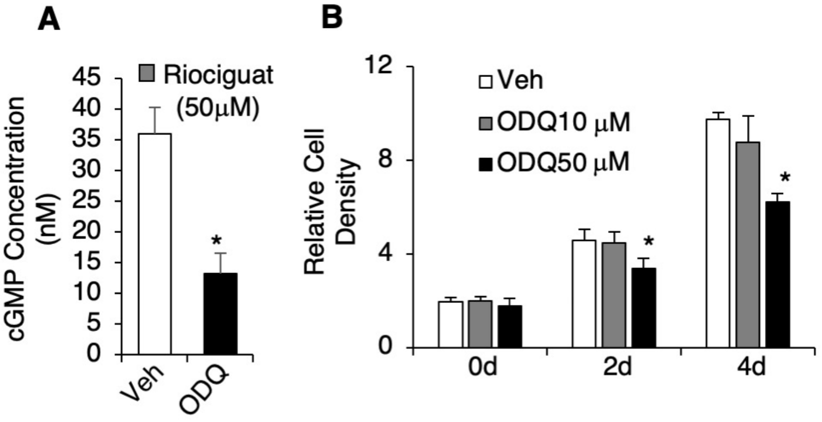

Supplementary Figure 7. sGC inhibitor ODQ decreased VCaP cell proliferation. (**A**) VCaP cells pretreated with riociguat were treated with sGC inhibitor ODQ (50μM) for 24h, followed by cGMP measurement. (**B**) VCaP cells treated with ODQ (0-50μM) for 0-4d were subjected to MUSE proliferation assay.

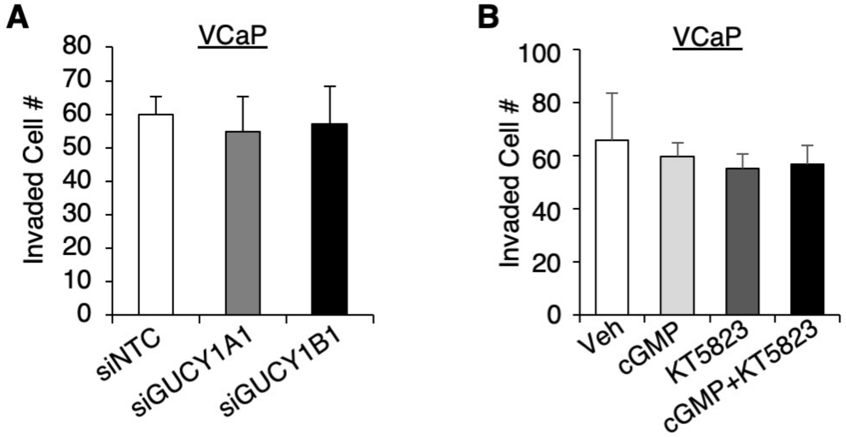

Supplementary Figure 8. sGC-cGMP signaling did not affect invasion capacity of VCaP cells. (**A**) VCaP cells transfected by siNTC versus siGUCY1A1 or siGUCY1B1 were subjected to Matrigel invasion assay. (**B**) VCaP cells treated with vehicle, 8-Br-cGMP (50μM), KT5823 (50μM), or 8-Br-cGMP plus KT5823 for 3d were subjected to Matrigel invasion assay. Cells invading through membrane were stained and quantified. Cell invasion was measured using the Cell Invasive Assay Kit from Chemicon following the manufacturer's protocol. Briefly, transfected VCaP cells suspensions in an upper chamber in serum-free medium were monitored for invasion into a lower chamber containing medium with 10% FBS. After 72 hours of incubation at 37°C, cells attached to the membrane of the lower chamber were stained.

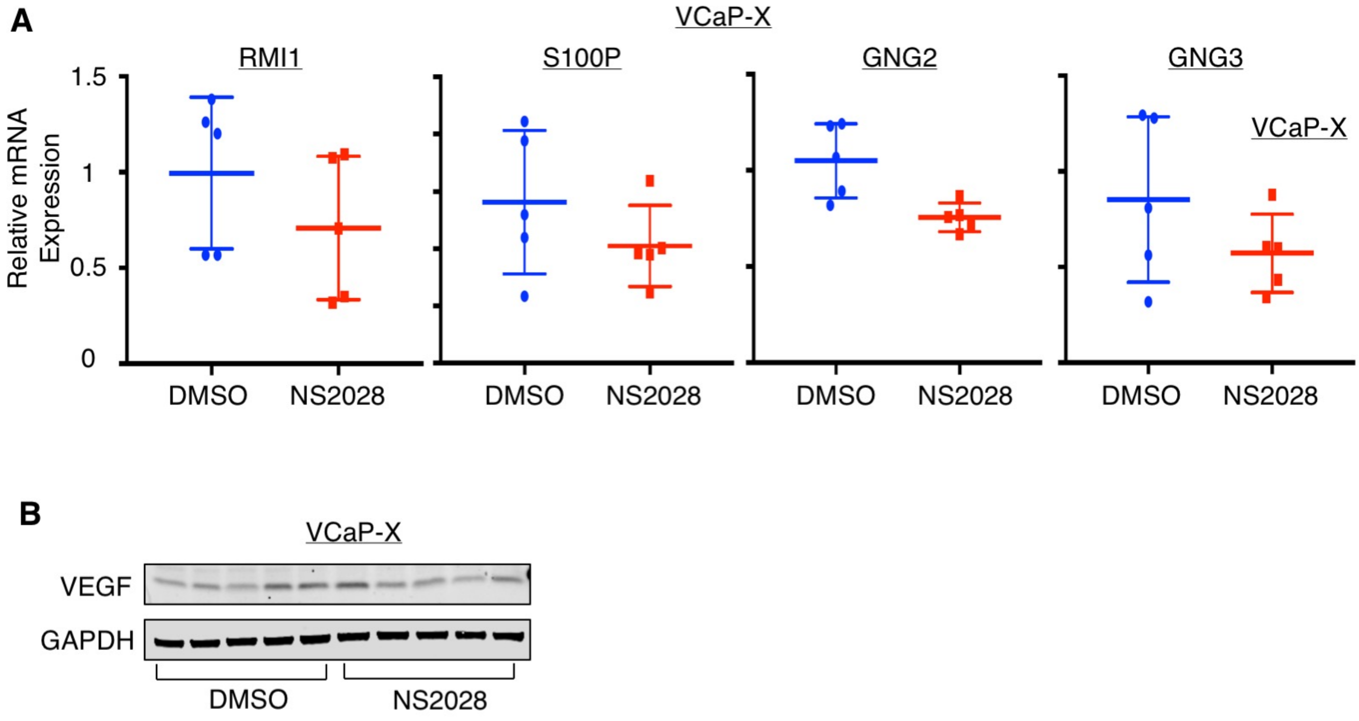

Supplementary Figure 9. NS2028 treatment suppressed the expression of GUCY1B1-regulated genes but did not decrease tumor angiogenesis in VCaP-derived xenograft model. (**A**, **B**) Intact SCID male mice bearing VCaP xenograft tumors received DMSO or NS2028 treatment for 19d and the tumor biopsies (N=5) were taken for (A) RT-PCR analyses and (B) immunoblotting for VEGF.

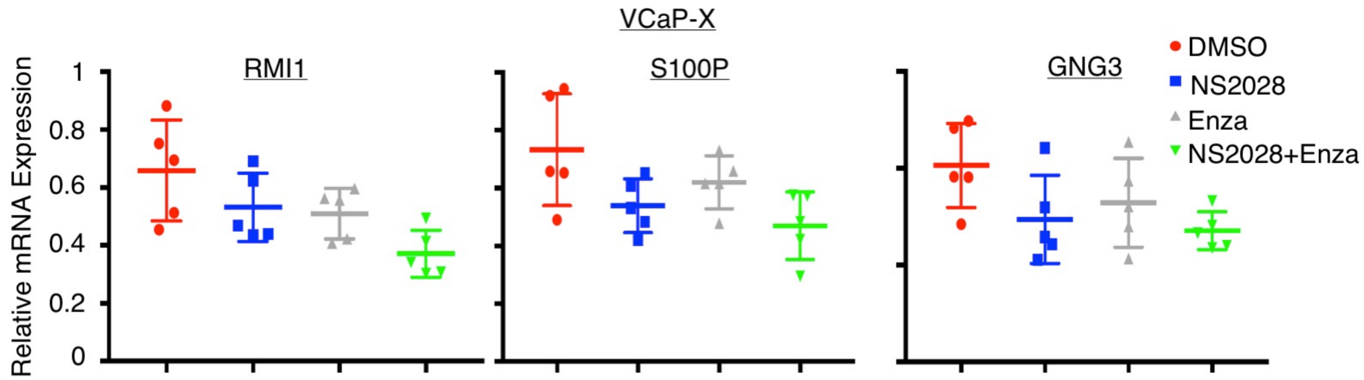

Supplementary Figure 10. The expression of GUCY1B1-regulated genes was repressed by the combined treatment of NS2028 and enzalutamide in VCaP-derived xenograft model. Intact SCID male mice bearing

VCaP xenograft tumors received the treatment of DMSO, NS2028 alone (50mg/kg), enzalutamide alone (10mg/kg), or in combination for 19d and the tumor biopsies (N=5) were taken for RT-PCR analyses.

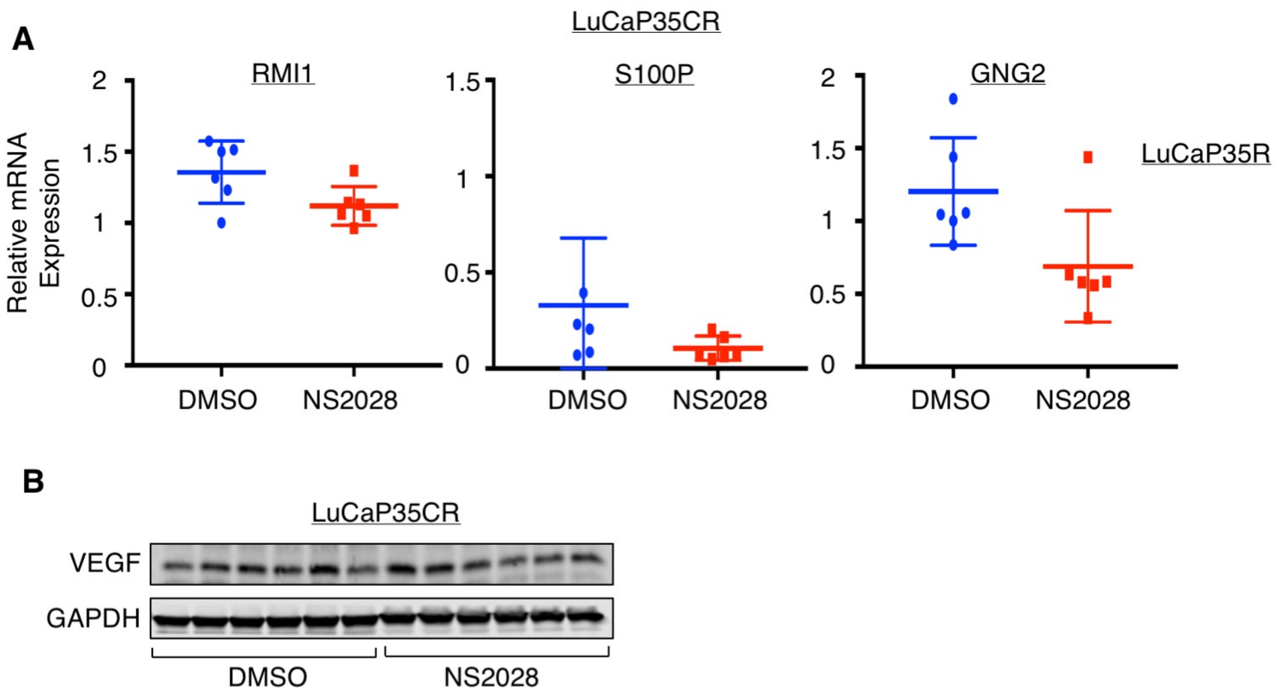

Supplementary Figure 11. NS2028 treatment suppressed the expression of GUCY1B1-regulated genes but did not decrease tumor angiogenesis in LuCaP35CR CRPC PDX model. (**A**, **B**) Castrated SCID male mice bearing LuCaP35CR xenograft tumors received DMSO or NS2028 treatment for 19d and the tumor biopsies (N=6) were taken for (A) RT-PCR analyses and (B) immunoblotting for VEGF.
